# Recyclable biomacromolecular hydrogel electrolytes for Zn-based aqueous batteries

**DOI:** 10.1093/nsr/nwaf383

**Published:** 2025-09-10

**Authors:** Tengsheng Zhang, Xinran Li, Dongyuan Zhao, Dongliang Chao

**Affiliations:** Laboratory of Advanced Materials, Aqueous Battery Center, Shanghai Key Laboratory of Molecular Catalysis and Innovative Materials, Collaborative Innovation Center of Chemistry for Energy Materials, Shanghai Wusong Laboratory of Materials Science, College of Smart Materials and Future Energy, Fudan University, China; Laboratory of Advanced Materials, Aqueous Battery Center, Shanghai Key Laboratory of Molecular Catalysis and Innovative Materials, Collaborative Innovation Center of Chemistry for Energy Materials, Shanghai Wusong Laboratory of Materials Science, College of Smart Materials and Future Energy, Fudan University, China; School of Physical and Mathematical Sciences, Nanyang Technological University, Singapore; Laboratory of Advanced Materials, Aqueous Battery Center, Shanghai Key Laboratory of Molecular Catalysis and Innovative Materials, Collaborative Innovation Center of Chemistry for Energy Materials, Shanghai Wusong Laboratory of Materials Science, College of Smart Materials and Future Energy, Fudan University, China; Laboratory of Advanced Materials, Aqueous Battery Center, Shanghai Key Laboratory of Molecular Catalysis and Innovative Materials, Collaborative Innovation Center of Chemistry for Energy Materials, Shanghai Wusong Laboratory of Materials Science, College of Smart Materials and Future Energy, Fudan University, China

Metallic Zn-based aqueous batteries (ZABs) have re-emerged as promising candidates for large-scale energy storage. However, their commercialization is still hindered by challenges such as uncontrolled zinc dendrite growth, hydrogen evolution and severe corrosion [1]. Conventional liquid electrolytes, although simple, are particularly susceptible to these issues [2]. To overcome these limitations, hydrogel polymer electrolytes (HPEs) have attracted growing interest. By suppressing free water activity, regulating Zn^2+^ flux and providing mechanical reinforcement against dendrites, HPEs can effectively mitigate these problems [3]. In addition, HPEs prevent electrolyte leakage and can simultaneously serve as separators, thereby simplifying cell configuration and imparting flexibility to ZABs [4].

However, the controllable fabrication of HPEs remains a significant challenge. Conventional hydrogels typically exhibit a trade-off between ionic conductivity and mechanical strength, excelling in one property at the expense of the other. Moreover, processing HPEs into thin films (<100 μm), which is essential for energy-dense cells, is particularly difficult. Sustainability further complicates the issue, as most petroleum-derived HPEs are neither recyclable nor environmentally friendly. Recently, Chen and co-workers reported in *National Science Review* a universal electrogelation strategy for the *in situ* construction of biomacromolecule-based hydrogel electrolytes. The resulting electrogelated chitosan hydrogel within 50 μm simultaneously achieves high mechanical strength (2.0–4.4 MPa), high ionic conductivity (10.1–19.5 mS cm^−1^) and excellent recyclability, thereby enabling reversible Zn plating/stripping and enhancing environmental sustainability [5].

Hydrogels are typically formed via sol–gel transitions, which can be triggered under various conditions to regulate both the gelation process and the resulting hydrogel structure. The choice and chemical nature of gelation triggers exert a profound influence on the success of the intended application. Numerous mechanisms have been reported, including temperature variation, component mixing, pH adjustment, redox-state alteration, enzyme addition, electromagnetic irradiation and ultrasound [6]. Nevertheless, for battery-related applications, these conventional approaches generally fail to achieve precise control over hydrogel thickness and homogeneity. In contrast, the potential-mediated pH variation strategy enables accurate *in situ* gelation, allowing controllable fabrication of hydrogel thickness and composition directly on the zinc anode surface (Fig. 1a). Compared with traditional chemical cross-linking methods, electrogelation offers unique advantages such as fast gelation, precise thickness control, uniform micro-/nanoporous structures, excellent geometric adaptability and scalability. Looking ahead, this electrogelation strategy could be extended beyond Zn to other multivalent metal systems (e.g. Mg and Al). Furthermore, its universal compatibility with diverse biopolymers underscores broad potential in tailoring electrochemical interfaces.

**Figure 1.**
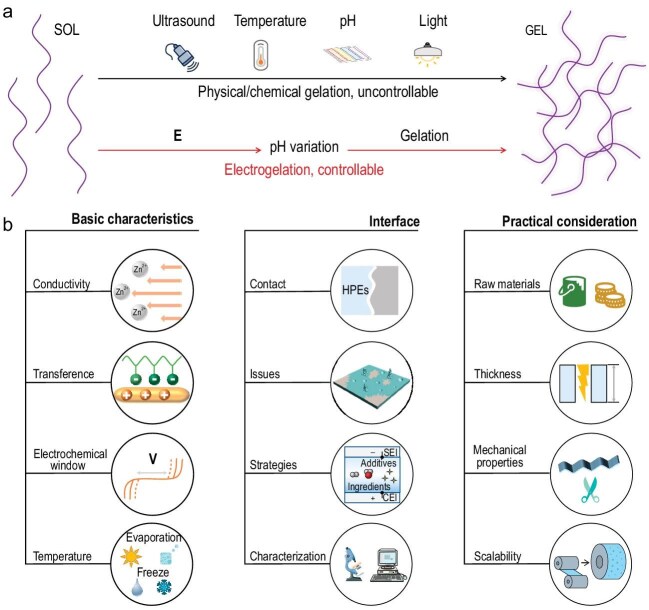
(a) Triggering conditions for hydrogel formation. (b) Main concerns in the development of HPEs.

It is worth noting that, unlike biomedical hydrogels, which have been extensively investigated and even commercialized [7], hydrogels for ZABs remain at a relatively early stage of development. For device-level applications, HPEs must simultaneously satisfy multiple requirements, including mechanical robustness, flexibility, thermal and chemical stability, as well as favorable electrochemical performance. Generally, HPEs can be categorized into non-cross-linked, physically cross-linked and chemically cross-linked networks. Non-cross-linked electrolytes typically suffer from poor mechanical integrity and often require the use of additional separators. By contrast, cross-linked hydrogels offer enhanced robustness and flexibility. For instance, a polyvinyl alcohol/Zn(CF_3_SO_3_)_2_ hydrogel electrolyte with physical cross-linking can autonomously self-heal via hydrogen bonding without external stimuli [8], while a chemically cross-linked polyacrylamide electrolyte exhibits good tensile strength (273 kPa) and remarkable stretchability of ≤3000% strain [9]. These examples demonstrate that cross-linked networks offer more stable structures, providing both mechanical strength and flexibility. Nevertheless, conventional HPEs are not without limitations. Due to the presence of water molecules in polymer networks, they inevitably freeze and lose elasticity at subzero temperatures, severely impairing their electrochemical performance and restricting their practical use in cold environments [10]. Proper modification strategies are therefore required to overcome these challenges under specific conditions. Moreover, beyond achieving better mechanical properties and electrochemical stability, large-scale manufacturability and cost control remain critical issues for the practical deployment of HPEs. In this regard, scalable preparation methods should be as simple as possible, while the raw materials must be abundant and low-cost to ensure economic feasibility.

Although HPEs have undergone significant advancements in recent years, their translation into practical battery systems still faces several key challenges. Future research should emphasize the following aspects (Fig. 1b).

***Optimization of fundamental electrolyte properties:*** The essential physicochemical parameters of HPEs require systematic improvement. Ionic conductivity, cation transference number, electrochemical stability window and thermal adaptability must be carefully optimized, as they directly determine overall battery performance. Striking a balance between high ionic conductivity and mechanical robustness is particularly important, while extending the electrochemical stability window to accommodate higher-voltage cathodes will further enhance energy density. In addition, achieving reliable performance across a broad temperature range from subzero to elevated conditions will be crucial for expanding the applicability of HPEs to diverse environments.

***Engineering stable electrode–electrolyte interfaces:*** The interface between hydrogels and electrodes remains a major bottleneck. Problems such as insufficient contact, parasitic reactions, corrosion and dendrite formation compromise long-term stability. Addressing these challenges calls for the rational modification of hydrogel compositions and the incorporation of functional additives to stabilize interfacial chemistry. At the same time, advanced *in situ* and *operando* characterization techniques should be applied to monitor interfacial evolution in real time. These approaches will not only clarify the mechanisms behind interfacial degradation, but also

provide guidance for the rational design of robust electrode–electrolyte interfaces.

***Practical considerations for device integration:*** For hydrogel electrolytes to be commercially viable, practical issues of raw material selection, salt chemistry, film thickness, mechanical integrity and scalable processing must be prioritized. Cost-effective and abundant raw materials will be essential to ensure economic feasibility. Minimizing hydrogel thickness is necessary to reduce internal resistance and increase energy density, highlighting the need for new fabrication strategies with precise thickness control. Furthermore, as HPEs usually serve dual roles as both electrolyte and separator, they must possess sufficient mechanical strength, elasticity and toughness to withstand operational stresses. Equally important is the scalability of fabrication methods; industrial roll-to-roll processing or other scalable techniques should be developed to meet the requirements of large-scale energy-storage applications.

In conclusion, the future development of HPEs will depend on an integrated approach that simultaneously tackles their intrinsic physicochemical properties, interfacial stability and practical manufacturability. By addressing these aspects in parallel, hydrogel electrolytes can move from laboratory-scale proof-of-concept systems to commercially deployable solutions, thereby enabling safer, more sustainable and higher-performance ZABs and beyond.

## References

[1] Zhang T, Tang Y, Guo S et al. Energy Environ Sci 2020; 13: 4625–65. 10.1039/D0EE02620D

[2] Wang X, Zhou W, Wang L et al. Adv Mater 2025; 37: 2501049. 10.1002/adma.202501049

[3] Lin Y, Huang J, Wang S et al. Adv Mater 2025; 25: 2509975. 10.1002/adma.202509975

[4] Sun Z, Bu F, Zhang Y et al. Angew Chem 2024; 136: e202402987. 10.1002/ange.202402987 38436516

[5] Huang J, Wang S, Yu L et al. Natl Sci Rev 2025; 12: nwaf308. 10.1093/nsr/nwaf308 40933453 PMC12418938

[6] Nele V, Wojciechowski JP, Armstrong JPK et al. Adv Funct Mater 2020; 30: 2002759. 10.1002/adfm.202002759

[7] Daly AC, Riley L, Segura T et al. Nat Rev Mater 2020; 5: 20–43. 10.1038/s41578-019-0148-6 34123409 PMC8191408

[8] Huang S, Wan F, Bi S et al. Angew Chem Int Ed 2019; 58: 4313–7. 10.1002/anie.201814653 30697965

[9] Li H, Liu Z, Liang G et al. ACS Nano 2018; 12: 3140–8. 10.1021/acsnano.7b09003 29589438

[10] Huang S, Hou L, Li T et al. Adv Mater 2022; 34: 2110140. 10.1002/adma.202110140 35122340

